# Response to the authors

**DOI:** 10.1186/s13613-020-00694-4

**Published:** 2020-06-08

**Authors:** Pauline de Jager, Martin C. J. Kneyber

**Affiliations:** 1 Department of Paediatrics, Division of Paediatric Critical Care Medicine, Beatrix Children’s Hospital, University Medical Center Groningen, University of Groningen, Groningen, The Netherlands; 2 Critical Care, Anaesthesiology, Perioperative & Emergency Medicine (CAPE), University of Groningen, Groningen, The Netherlands

We like to thank the authors for their interest in our manuscript and their positive feedback. High-frequency oscillatory ventilation (HFOV) is used in our unit for any type of PARDS when the patient meets specific criteria as outlined in our manuscript (in summary, peak inspiratory pressure [PIP] > 28–32 cm H_2_O, PEEP > 8 cm H_2_O, FiO_2_ > 0.60, and oxygenation index [OI] increases on three consecutive 1-h measurements despite increasing PEEP) [1]. We understand the author’s perspective that HFOV might be more effective in certain types of PARDS, but we advocate that HFOV should not only be considered in case of refractory hypoxaemia, but also when the bedside team wants to prevent ventilator settings becoming toxic. An individualised lung volume optimisation manoeuvre (such as the staircase incremental–decremental titration of the continuous distending pressure (CDP) helps in identifying patients who have potential for lung recruitability since the response is highly heterogeneous among PARDS [2]. As our data showed, such an individualised manoeuvre can be tolerated well in terms of haemodynamic effects with a minimal risk of barotrauma (in fact, we observed no barotraumas following the manoeuvre in our cohort).

The authors raise an important point: what is the “optimal” frequency in relation to PARDS severity? Although the concept of the corner frequency is quite clear, it is difficult to detect at the bedside how the “optimal” frequency can be identified in heterogenous PARDS [3]. Basically, the lower the lung compliance, the higher the frequency probably should be. For simplicity, when we implemented the HFOV clinical algorithm in our unit, the advice was to start with 12 Hz in all patients, irrespective of age or PARDS severity and titrate immediately after the lung volume optimisation manoeuvre using the PCO_2_ to give direction (e.g. frequency up or down). Our data confirmed that it was possible to do this in all patients, irrespective of age (Fig. 1).

**Fig. 1:**
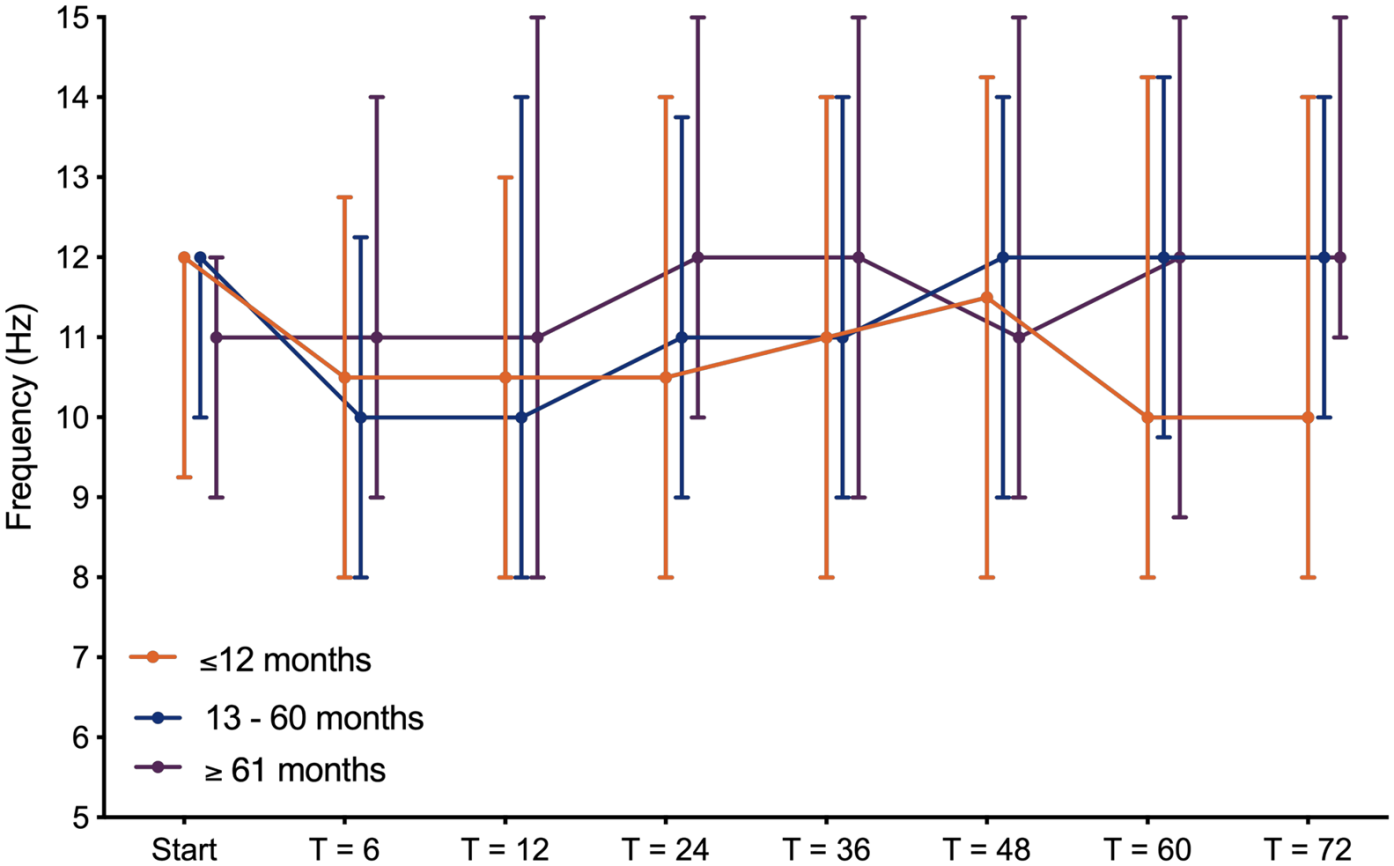
Level and time course of achieved frequency (*F*) during the first 72 h of high-frequency oscillatory ventilation (HFOV), stratified by age. “Start” is the first measurement immediately after the recruitment manoeuvre. Data are depicted as median (25–75 interquartile range). * Denotes *p* < 0.05

We agree that in a subgroup of patients in our cohort, especially those with mild-to-moderate PARDS optimisation of conventional mechanical ventilation settings might have been attempted. The median OI of 38 as pointed out by the reviewer is the OI *after* the lung volume optimisation manoeuvre, hence the high CDP we use as part of the open-lung concept confounds the OI. It is true that in general in the paediatric intensive care unit there is a relatively low use of positive end-expiratory pressure (PEEP) and tolerance of high FiO_2_ instead. However, the best strategy to optimise CMV in children with severe PARDS remains uncertain [4]. To date, there is no specific PEEP strategy shown to be beneficial nor are there outcome data demonstrating that higher PEEP is better than lower PEEP in PARDS, although there are some suggestions that lower PEEP in PARDS may be associated with increased mortality [5]. We also do not know what the optimal Vt is in (severe) PARDS [6]. Hence, we advocate that HFOV should also be considered if the bedside team wants to prevent ventilator settings becoming toxic.

We eagerly await the results of a 2-by-2 factorial randomised controlled trial comparing the effects of ventilation strategy (CMV vs HFOV) with or without prone positioning (http://www.prospect-network.org) on patient outcome [7].

## Data Availability

Not applicable.

## References

[1] de Jager P, Kamp T, Dijkstra SK, Burgerhof JGM, Markhorst DG, Curley MAQ (2019). Feasibility of an alternative, physiologic, individualized open-lung approach to high-frequency oscillatory ventilation in children. Ann Intensive Care..

[2] de Jager P, Burgerhof JGM, Koopman AA, Markhorst DG, Kneyber MCJ (2019). Lung volume optimization maneuver responses in pediatric high frequency oscillatory ventilation. Am J Respir Crit Care Med..

[3] Venegas JG, Fredberg JJ (1994). Understanding the pressure cost of ventilation: why does high-frequency ventilation work?. Crit Care Med.

[4] Kneyber MCJ, de Luca D, Calderini E, Jarreau PH, Javouhey E, Lopez-Herce J (2017). Recommendations for mechanical ventilation of critically ill children from the Paediatric Mechanical Ventilation Consensus Conference (PEMVECC). Intensive Care Med.

[5] Khemani RG, Parvathaneni K, Yehya N, Bhalla AK, Thomas NJ, Newth CJL (2018). PEEP lower than the ARDS network protocol is associated with higher pediatric ARDS mortality. Am J Respir Crit Care Med..

[6] de Jager P, Burgerhof JG, van Heerde M, Albers MJ, Markhorst DG, Kneyber MC (2014). Tidal volume and mortality in mechanically ventilated children: a systematic review and meta-analysis of observational studies*. Crit Care Med.

[7] Kneyber MCJ, Cheifetz IM, Curley MAQ (2020). High-frequency oscillatory ventilation for PARDS: awaiting PROSPect. Crit Care.

